# An arrestin-1 surface opposite of its interface with photoactivated rhodopsin engages with enolase-1

**DOI:** 10.1074/jbc.RA120.013043

**Published:** 2020-04-01

**Authors:** Connie Jaqueline Miranda, Nicole Fernandez, Nader Kamel, Daniel Turner, Del Benzenhafer, Susan N. Bolch, Jacob T. Andring, Robert McKenna, W. Clay Smith

**Affiliations:** ‡ Department of Ophthalmology, University of Florida, Gainesville, Florida 32610; § Department of Biochemistry and Molecular Biology, University of Florida, Gainesville, Florida 32610

**Keywords:** arrestin, glycolysis, photoreceptor, phototransduction, protein complex, G protein-coupled receptor (GPCR), protein conformation, enolase, fluorescence quench protection, light sensing

## Abstract

Arrestin-1 is the arrestin family member responsible for inactivation of the G protein–coupled receptor rhodopsin in photoreceptors. Arrestin-1 is also well-known to interact with additional protein partners and to affect other signaling cascades beyond phototransduction. In this study, we investigated one of these alternative arrestin-1 binding partners, the glycolysis enzyme enolase-1, to map the molecular contact sites between these two proteins and investigate how the binding of arrestin-1 affects the catalytic activity of enolase-1. Using fluorescence quench protection of strategically placed fluorophores on the arrestin-1 surface, we observed that arrestin-1 primarily engages enolase-1 along a surface that is opposite of the side of arrestin-1 that binds photoactivated rhodopsin. Using this information, we developed a molecular model of the arrestin-1–enolase-1 complex, which was validated by targeted substitutions of charge-pair interactions. Finally, we identified the likely source of arrestin's modulation of enolase-1 catalysis, showing that selective substitution of two amino acids in arrestin-1 can completely remove its effect on enolase-1 activity while still remaining bound to enolase-1. These findings open up opportunities for examining the functional effects of arrestin-1 on enolase-1 activity in photoreceptors and their surrounding cells.

## Introduction

The arrestin family of proteins is well-established as a key regulator of G protein–coupled receptors, functioning in both their desensitization and intracellular trafficking (see Ref. 1 for a recent review). In the vertebrate visual system, arrestin-1 desensitizes the visual pigment in both rod and cone photoreceptors, binding light-activated and phosphorylated rhodopsin to sterically occlude transducin (2–4). Although there is no evidence that rhodopsin is internalized, and thus no role for arrestin-1 in rhodopsin intracellular trafficking, a number of studies have identified additional interactions for arrestin-1 other than rhodopsin desensitization (5). This list of interactions includes arrestin-1 binding calcium-calmodulin, potentially buffering changes in cytosolic calcium levels in photoreceptors (6); interaction with Src family tyrosine kinases for activation of extracellular signal–regulated kinase 1/2 and E3 ubiquitin ligases (7); and interaction with *N*-ethylmaleimide–sensitive factor (NSF)^4^ for regulation of synaptic signaling (8).

In addition to these binding partners for arrestin-1, there is also an interaction between arrestin-1 and enolase-1 (9), one of the key enzymes in the glycolysis pathway that catalyzes the interconversion of 2-phosphoglycerate to phosphoenolpyruvate. This interaction is specific for enolase-1, an observation that is surprising because enolase-2 is considered to be the “neuronal” enolase (10). Intriguingly, the binding of arrestin-1 to enolase-1 affects the catalytic activity of enolase, reducing its rate of activity by ∼25%. Although a reduction of 25% activity may not seem like a large change, photoreceptors require on the order of 10^5^ ATP molecules/s/cell, ranking them as one of the highest energy-consuming cells in the body (11). Furthermore, photoreceptors metabolize 80–96% of available glucose into lactic acid via aerobic glycolysis (12, 13), returning the lactate byproduct as an essential metabolic component to the retinal pigmented epithelium and Müller glia. Consequently, small changes in glycolytic efficiency could have a large impact on photoreceptors because of their extreme energetic demands. Because of the impact of arrestin-1 on enolase-1 catalysis, we initiated this study to develop a molecular understanding of the interaction between arrestin-1 and enolase-1 with the goal being to understand how the binding of arrestin-1 could affect the catalytic activity of enolase-1.

## Results

In a previous study, we identified that arrestin-1 selectively interacts with enolase-1 in photoreceptors, modulating the catalytic activity of enolase-1 (9). In this study, we investigated the biophysical nature of this interaction between arrestin-1 and enolase-1 with the goal being to understand the mechanism for how the binding of arrestin-1 could affect the enzymatic activity of enolase-1. As a first step toward understanding the interaction of these two proteins, we used targeted fluorescence labeling of arrestin-1 and fluorescence quenching to map the surface on arrestin-1 with which enolase-1 interacts. For this study, 28 cysteine substitutions were introduced individually into arrestin-1 at residues that are positioned across the surface of arrestin-1 (Table 1). Importantly, these cysteine substitutions were introduced into an arrestin-1 in which native Cys-63 and Cys-143 were converted to alanine to remove the endogenous reactive cysteines in arrestin-1. These introduced cysteine residues were labeled with the thiol-reactive fluorophore monobromobimane (mBBr).

**Table 1:** **Fluorescence quenching of mBBr-labeled arrestin-1 cysteine mutants by potassium iodide with protection by enolase-1**

Cysteine substitution	*F*_(A)_	*F*_(A + K)_	*F*_(A + E + K)_	*P*_f_
H10C	12,595.7	1,449.61	10,279.5	0.792
R18C	40,060.9	1,148.8	963.2	−0.005
Y25C	17,932.1	789.91	599.8	−0.011
K28C	23,275.8	557.8	17,195.5	0.732
R37C	28,962.6	682.3	16,642.6	0.564
E50C	25,319.4	891.0	8,143.2	0.297
K53C	34,263.2	421.3	11,031.5	0.314
I72C	40,304.8	549.0	630.0	0.002
S86C	20,069.2	5,060.5	6,159.6	0.073
V94C	16,472.6	935.5	1,901.7	0.062
A113C	25,323.9	750.9	3,465.0	0.109
Y125C	44,782.9	13,617.9	13,978.0	0.012
V139C	27,086.9	813.6	778.3	−0.001
K166C	24,572.6	404.6	1,041.5	0.026
D183C	24,307.5	538.9	19,234.9	0.787
R189C	23,296.8	490.8	11,133.8	0.467
W194C	44,782.9	13,617.9	13,978.0	0.012
S199C	23,297.2	662.5	636.1	−0.001
S210C	13,660.6	724.1	8,717.0	0.618
E218C	8,846.4	953.6	9,498.0	1.083
E231C	6,973.8	682.0	694.6	0.002
S251C	6,123.7	674.5	730.5	0.011
K267C	5,523.2	350.3	2,851.8	0.484
V281C	6,002.9	1,167.2	2,439.3	0.269
E302C	4,562.3	686.6	3,676.4	0.772
D317C	33,509.8	11,518.1	11,514.0	0.002
D362C	4,735.3	950.7	5,491.7	1.200
E393C	3,604.6	542.6	952.8	0.134

We next optimized the conditions for analysis of quenching of the fluorophore by potassium iodide (KI) (14). First, we identified the optimum KI concentration for quenching, performing a titration assay using three different cysteine-substituted arrestin-1s (K53C, S86C, and Y125C) labeled with mBBr, quenching with increasing concentrations of KI. For all three labeled positions, 50% quench of fluorescence of the 3 μm arrestin-bimane was achieved at ∼33 mm KI or with a 10^4^ molar excess of KI over the bimane-labeled arrestin.

We next titrated the concentration of enolase-1 that could provide protection of mBBr fluorescence from quenching by the KI, determining that a 90% quench protection could be obtained with a 20-fold molar excess of enolase-1 to arrestin-1 for H10C labeled with mBBr.

**Figure 1.**
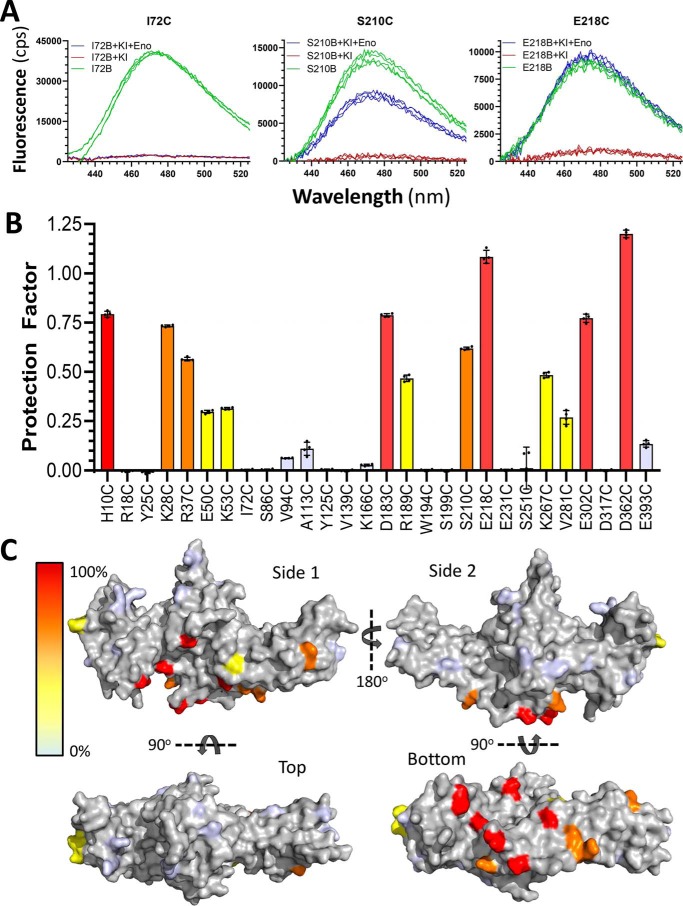
**Protection of fluorescence quenching of bimane-labeled arrestin-1 by enolase-1.** *A*, fluorescence emission spectra from examples of three arrestin-1 mutants labeled with bimane (*green traces*) and the quenching of fluorescence caused by potassium iodide in the presence (*blue traces*) or absence (*red traces*) of a 20-fold molar excess of enolase-1; enolase-1 provides no protection of the bimane fluorophore for I72C, intermediate protection for S210C, and full protection for E218C. *B*, summary of the quenching protection provided by enolase-1 for 28 cysteine-substituted mutants of arrestin-1 (as indicated) labeled with mBBr (each *bar* shows mean ± S.D. (*error bars*); *n* = 4). Mutants for which enolase provided >75% protection of the KI quenching are shown in *red*, 50–75% protection is shown in *orange*, 25–49% protection is shown in *yellow*, and 0–24% protection is shown in *pale blue. C*, the various mutants indicated in *B* are shown plotted on a three-dimensional rendering of arrestin-1, retaining the same *color coding* for amino acids as in *B*; the four models show four views of the same rendering (two sides, top, and bottom).

Using these parameters of ∼50% quench with a 10^4^ molar excess of KI over arrestin-1 and 20-fold molar excess of enolase-1 over arrestin-1, we scanned the arrestin-1 cysteine point mutations for the potential of enolase-1 to protect the mBBr-labeled arrestin-1 for fluorescence quenching. For this analysis, we measured the fluorescence emission of the mBBr-labeled arrestin-1 with and without potassium iodide to determine the range of quenching by KI and measured the fluorescence emission quench by KI with and without enolase-1. Three examples of the range of quench protection are shown (Fig. 1*A*), with enolase-1 providing no protection of the mBBr on I72C, ∼60% protection for S210C, and essentially complete protection for E218C. For each of the 28 labeled arrestin-1 proteins, a quench protection factor was calculated as described under “Experimental procedures” (Table 1 and Fig. 1*B*). This quench protection analysis revealed a range of residues that when labeled by mBBr were highly protected from quenching by enolase. When mapped onto the surface of the arrestin-1 structure, these residues largely mapped to a single surface along the “bottom” of the arrestin-1 protein (Fig. 1*C*).

**Figure 2.**
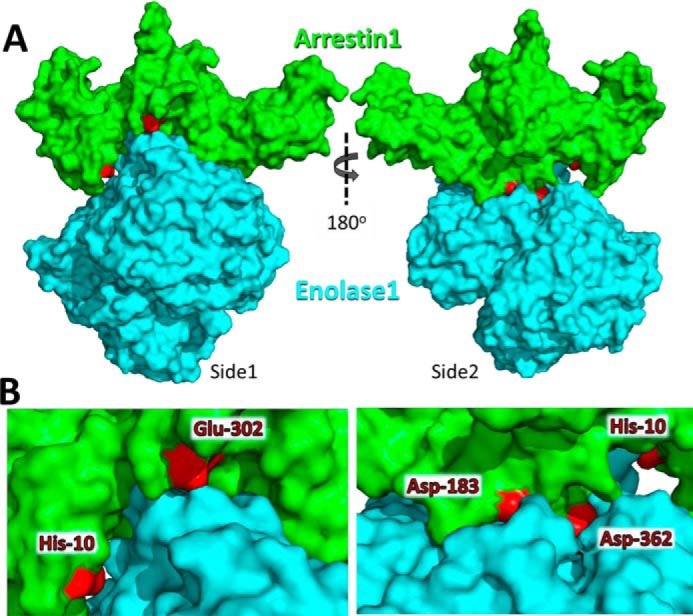
**Molecular model of arrestin-1 (*green*) docked with a dimer of enolase-1 (cyan).** *A*, docking model showing opposite sides of the same model, with arrestin-1 residues His-10, Asp-183, Glu-218, Glu-302, and Asp-362 indicated in *red. B*, *magnified view* of the interface area as shown in *A*.

We then used this information to generate a molecular model of the interaction between arrestin-1 and enolase-1, performing energy minimization docking of arrestin-1 with an enolase-1 dimer using ClusPro 2.0. A dimer of enolase-1 was used for this model because this is the typical physiological state of enolase-1 (15), and our previous study had shown that arrestin-1 cross-links to a dimer of enolase-1 (9). Based on the fluorescence quench studies, the interaction sites were constrained to include residues His-10, Asp-183, Glu-218, Glu-302, and Asp-362 of arrestin-1. The resulting model of the complex predicted both N- and C-terminal domains of arrestin-1 to bind to a single unit of the enolase-1 dimer (Fig. 2), forming numerous interactions with surface residues, including several paired-charge interactions.

**Figure 3.**
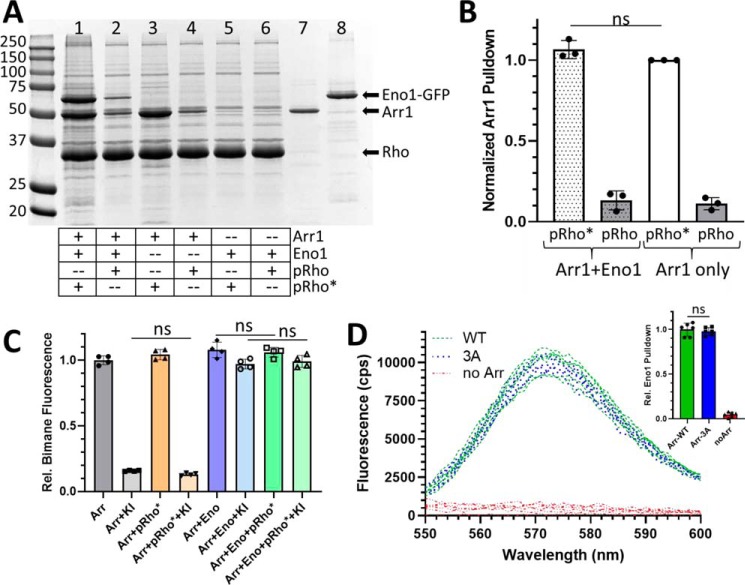
**Enolase-1 binding is not affected by arrestin-1 conformational changes induced by binding to rhodopsin or by mutations that mobilize the C terminus of arrestin-1.** *A*, arrestin-1 was pulled down by phosphorylated rhodopsin kept in the dark (*pRho*) or exposed to light (pRho*) in rod photoreceptor disc membranes, either in the presence (*lanes 1* and *2*) or absence of enolase-1/GFP (*lanes 3* and *4*). Enolase-1/GFP pulled down with arrestin-1 only when arrestin-1 pulled down with pRho* (*lane 1*) and not when arrestin-1 was absent (*lanes 5* and *6*). *Lanes 7* and *8* show aliquots of the purified arrestin-1 (*Arr1*) and enolase-1/GFP (*Eno1-GFP*), respectively, used in the pulldown assay. The gel shows protein samples separated by 12% SDS-PAGE and stained with Coomassie Blue; molecular mass markers are shown in kilodaltons. *B*, quantitative summary of arrestin-1 pulled down with phosphorhodopsin kept in the dark (*pRho*) or activated by exposure to light (pRho*) in the presence of equimolar enolase-1 (*hatched bars*) or without enolase-1 (*unfilled bars*); *bars* show mean ± S.D. (*error bars*) (*n* = 3); *ns*, no significant change. *C*, E218C-mBBr–labeled arrestin-1 quenching by potassium iodide (*gray bars*) is not changed by binding of arrestin-1 to pRho* (*orange bars*); similarly, protection of bimane quenching provided by enolase-1 (*blue bars*) is also not affected by arrestin-1 binding to pRho* (*green bars*); *bars* show mean ± S.D. (*error bars*) (*n* = 4); *ns*, no significant change. *D*, WT arrestin-1 (*green curve*) or “3A” arrestin-1 (F375A/V376A/F377A; *blue curves*) was immunoprecipitated with anti-arrestin-1 antibody, pulling down enolase-1 fluorescently labeled with Alexa Fluor 546. The *curves* show the fluorescent profile of the captured enolase-1 in replicate experiments, compared with the background of enolase-1 capture when no arrestin-1 is present (*red curves*); the *inset* shows a quantitative summary of the pulldown assay, normalized to the WT arrestin-1; *bars* show mean ± S.D. (*error bars*) (*n* = 6); *ns*, no significant change.

Because the surface of the arrestin-1 that is modeled to interact with enolase-1 is on the side opposite of arrestin-1 that engages rhodopsin (16–18), we tested whether the binding of arrestin-1 to light-activated, phosphorylated rhodopsin (pRho*) would exclude enolase-1 binding. For this experiment, we used phosphorylated rhodopsin prepared in rod disc membranes and performed co-sedimentation analysis of arrestin-1 with and without enolase-1/GFP after light activation of the rhodopsin (Fig. 3*A*). Note that for this experiment, we used an enolase-1/GFP fusion so that the molecular masses of arrestin-1 and enolase-1 could be distinguished on gel electrophoresis. In this analysis, enolase-1/GFP co-sedimented with arrestin-1 on membranes with pRho* (Fig. 3*A*, *lane 1*), but not in mixtures that contained no arrestin-1 (*lane 5*) or in which the phosphorhodopsin was not light-activated (*lanes 2* and *6*), indicating that arrestin-1 binding to rhodopsin does not exclude the interaction of arrestin-1 with enolase-1. Conversely, quantitative analysis of the arrestin-1 pulled down with pRho* in the presence or absence of enolase-1/GFP indicates that the binding of enolase-1 to arrestin-1 does not affect the binding of arrestin-1 to pRho* (Fig. 3*B*).

As an alternative way to examine the simultaneous interaction of arrestin-1 with both pRho* and enolase-1, we monitored whether the quenching protection provided by enolase-1 for arrestin-1 labeled with mBBr at E218C was affected by arrestin-1 binding to pRho*. When E218C-mBBr is mixed with pRho*, there is no protection of the mBBr fluorophore from quenching by potassium iodide (Fig. 3*C*, *orange bars*). In contrast, enolase-1 provides nearly complete protection of the fluorophore either without pRho* (*blue bars*) or with arrestin-1 bound to pRho* (*green bars*). These studies suggest not only that arrestin-1 can simultaneously engage enolase-1 and pRho*, but also that binding of enolase-1 to arrestin-1 is independent of whether arrestin-1 is in its inactive or receptor-bound conformation.

As a further test of this observation, we also performed a co-precipitation assay of enolase-1 by immunoprecipitating arrestin-1 with anti-arrestin-1 antibody on Protein G-coated magnetic beads, and measuring pulldown of enolase-1 that was fluorescently labeled with Alexa Fluor 546. For this assay, we used either native arrestin-1 (WT) as the inactive form or arrestin-1 with the so called “3A” mutations (*i.e.* F375A/V376A/F377A), which mobilizes the arrestin-1 C terminus, allowing it to adopt an active conformation that does not require rhodopsin phosphorylation to bind (19, 20). In this assay, there was no distinguishable difference in the pulldown of labeled enolase-1 between WT and the preactivated arrestin-1 (Fig. 3*D*).

**Figure 4.**
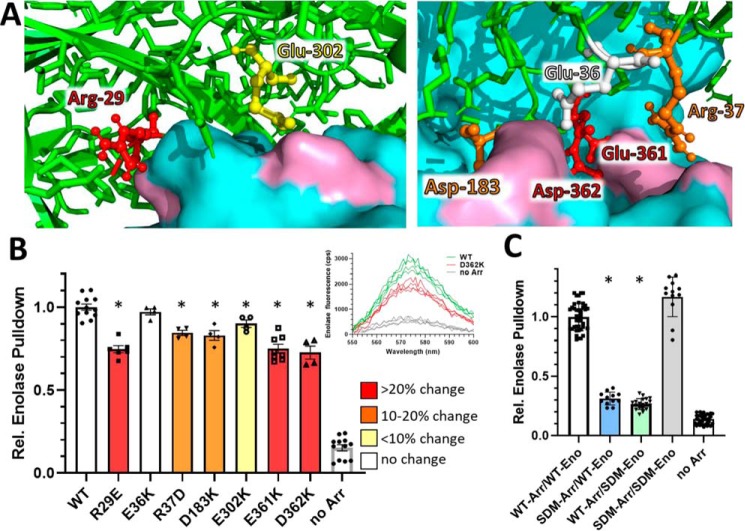
**Enolase-1 pulldown by mutants of arrestin-1 designed to disrupt charge-pair interactions with enolase-1.** *A*, molecular model of the arrestin-1/enolase-1 complex showing the charged residues on arrestin-1 (*ball and stick*) that were selected for reversal and their proximity to enolase-1 (*cyan*, surface representation, with enolase-1 charged pair shown in *pink*). *B*, arrestin-1 with the indicated point mutations was immunoprecipitated with an anti-arrestin mAb attached to magnetic beads, pulling down fluorescently labeled enolase-1. The captured enolase-1 is normalized to the pulldown of enolase-1 by arrestin-1 with no mutations (WT); the *bar* indicated as *no Arr* shows the background pulldown of labeled enolase-1 in the absence of any arrestin-1; the *inset* shows examples of raw emission spectra collected for D362K mutant. *C*, enolase-1 pulldown with arrestin-1 with all seven point mutations (*SDM-Arr*; *blue bar*) or arrestin-1 pulldown of enolase-1 with all seven charge-pair mutation (*SDM-Eno*; *green bar*) showed essentially no pulldown. Combining SDM-Arr with SDM-Eno restored pulldown of enolase (*gray bar*); enolase-1 pulldowns by the mutant arrestins that are significantly different from WT are indicated with an *asterisk* (*p* < 0.05).

Returning to our model of the arrestin-1/enolase-1 complex, we next wanted to further validate this model so that we could then use it for predictive studies. We elected to use the charge-pair interactions to empirically validate the modeled complex. Seven charge-pair interactions (Table 2 and Fig. 4*A*) were identified from the model as having the closest interactions. These seven residues were singly mutated on the arrestin-1 protein to reverse the charge of the amino acid side chain while approximately preserving side-chain size (*e.g.* Glu to Lys or Arg to Asp). Following heterologous expression and purification, each arrestin-1 mutant was then assessed for its interaction with enolase-1, using the anti-arrestin-1 immunoprecipitation assay described above to pull down fluorescently labeled enolase-1. In this assay, mutations R29E, E361K, and D362K reduced the pulldown of enolase-1 by ∼25% (Fig. 4*B*). Mutations R37D, D183K, and E302K had a smaller, but also significant, effect on interaction with enolase-1. The significant effect of each of these amino acid substitutions, with the exception of E36K, on the interaction with enolase-1 suggests that the model of interaction between arrestin-1 and enolase-1 (Fig. 2) is correct.

**Table 2:** **Charged-pair interactions between arrestin-1 and enolase-1 in the energy-minimized docking model shown in Fig. 4** The amino acid changes made for charge reversal in the various arrestin-1 and enolase-1 mutants are indicated in parentheses after each amino acid.

Arr1 residue (charge reversal)	Enolase-1 residue (charge reversal)	Molecular distance
		Å
Arg-29 (Glu)	Asp-265 (Lys)	2.8
Glu-36 (Lys)	Lys-53 (Glu)	2.7
Arg-37 (Asp)	Glu-197 (Lys)	1.9
Asp-183 (Lys)	Lys-59 (Asp)	2.3
Glu-302 (Lys)	Lys-255 (Asp)	1.8
Gu-361 (Lys)	Lys-196 (Glu)	2.6
Asp-362 (Lys)	Lys-192 (Glu)	2.5

Because each mutation partially disrupted the interaction between arrestin-1 and enolase-1, we reasoned that all seven mutations together would likely have a more significant effect. Accordingly, we assembled all seven point mutations into a single arrestin-1 protein and also assembled the corresponding charge reversals in the enolase-1 molecule. When used in our pulldown assay, these mutagenized proteins decreased the binding to their WT counterpart by more than 80% (Fig. 4*C*). Importantly, when the combination mutant of arrestin-1 is paired with the combination mutant of enolase-1, binding interaction is restored (Fig. 4*C*, *gray bar*), providing compelling evidence that these mutations are occurring on complementary pairs of interactions.

**Figure 5.**
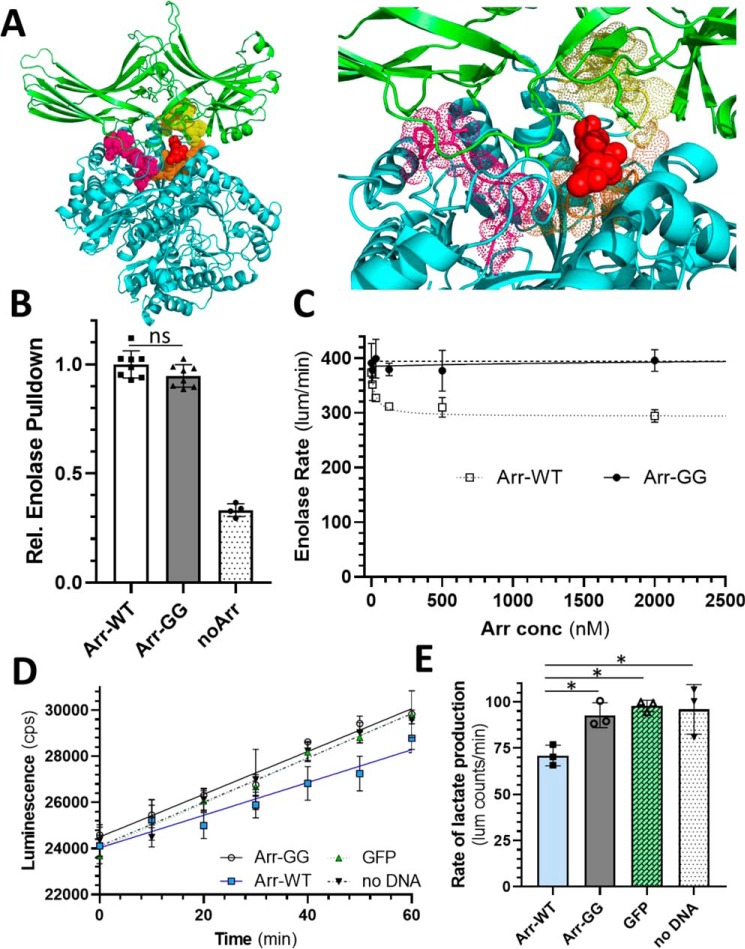
**Arr-E361G/D362G binds enolase-1 but does not affect enolase catalytic activity.** *A*, molecular model of the Arr1-Eno1 complex showing the proximity of Glu-361/Asp-362 on arrestin-1 (*red spheres*) relative to active site loops L1 (*magenta dots*), L2 (*orange dots*), and L3 (*yellow dots*) on enolase-1. *B*, immunoprecipitation pulldown of fluorescently labeled enolase-1 was performed using native arrestin-1 (*Arr-WT*) or arrestin-1 with E361G and D362G mutations (*Arr-GG*). The binding of enolase-1 was not significantly changed by the double mutations E361G/D362G; *bars* show means ± S.D. (*error bars*) (*n* = 8). *C*, the influence of these same two mutations on enolase-1 catalytic activity was assessed, monitoring the production of ATP from the processing of 2-phosphoglycerate to pyruvate. The E361G/D362G arrestin-1 (*closed circles*) did not show inhibition of enolase-1 activity that is evident with native arrestin-1 (*open squares*); each *point* shows mean ± S.D. (*error bars*) (*n* = 3). *D*, lactate production from HEK-293T cells transfected with plasmids expressing arrestin-1 (*Arr-WT*; *blue squares*), arrestin-1 with E361G/D362G (*Arr-GG*; *open circles*), GFP (*green triangles*), or untransfected (*no DNA*; *inverted triangles*); lines show linear regression through mean ± S.D. (*error bars*) (*n* = 3). *E*, rates of lactate production determined from *D*; *bars*, mean ± S.D. (*n* = 3); significantly different rates are indicated with an *asterisk* (*p* < 0.05).

Because these binding studies indicated that our model of the interaction between arrestin-1 and enolase-1 is accurate, we next pursued the goal of determining the structural mechanism for how arrestin-1 binding to enolase-1 can affect the catalytic activity of enolase-1. Our previous study demonstrated that the binding of arrestin-1 to enolase-1 diminished the catalytic rate of enolase-1 by ∼25% (9). Accordingly, we examined our model of the interaction, focusing on enolase-1 loops L1, L2, and L3 and the magnesium coordinating residues Ser-36, Asp-244, Glu-292, and Asp-317, which comprise the key components of the enolase-1 active site (21, 22). In our model, arrestin-1 residues Glu-361 and Asp-362 are the residues most deeply inserted into enolase-1, and these two residues are also in close proximity to all three of the loops that comprise one of the active sites in the enolase-1 dimer, particularly Ser-156 and Gly-159 in loop L2 (Fig. 5*A*). Because we previously demonstrated that the binding of arrestin-1 to enolase-1 diminished the catalytic rate of enolase-1 by ∼25%, we speculated that this interaction point might be the source of the perturbation, likely by steric interference. To investigate this idea, we mutagenized both Glu-361 and Asp-362 to glycine, reasoning that if a steric-hindrance effect was occurring, these substitutions would potentially relieve the interference. We first examined whether these mutations affected the binding of arrestin-1 to enolase-1 using the same pulldown assay of fluorescently labeled enolase-1 described previously. In this assay, the pulldown of enolase-1 with the E361G/D362G double mutant of arrestin-1 is indistinguishable from that of native arrestin-1 (Fig. 5*B*). This finding indicates that these two mutations do not significantly disrupt the overall interaction between arrestin-1 and enolase-1. We then moved on to determine whether these mutations influenced the impact of arrestin-1 on enolase-1 catalytic activity. In this assay, the catalytic activity of enolase was measured by monitoring the production of ATP from the processing of 2-phosphoglycerate to pyruvate (Fig. 5*C*). WT arrestin-1 inhibited the catalytic activity of enolase-1 by ∼25%. Significantly, this inhibition of enolase catalysis was completely absent in the E361G/D362G double mutant of arrestin-1, even at a 320-fold molar excess. This finding suggests that Glu-361 and Asp-362 are largely responsible for altering the catalytic activity of enolase-1 when the complex forms.

To determine whether the effect of arrestin-1 on enolase catalysis has any potential physiological function, we monitored the glycolytic output of lactate from HEK-293T cells that had been transfected with arrestin-1 (Fig. 5*D*). Because HEK cells use a combination of glycolysis and oxidative phosphorylation for energy production, we biased the cells toward glycolysis by inhibiting oxidative phosphorylation using rotenone and antimycin A to block complex I and complex III of the respiratory chain (23). Measurement of lactate produced by these cells showed a significant reduction in the cells that were transfected with arrestin-1 (Fig. 5, *D* and *E*) compared with cells that were either untransfected or transfected with a plasmid expressing GFP. This result indicates that arrestin-1 can affect glycolytic output in a tissue culture context. Consistent with our previous observation, transfection of the modified arrestin-1 with the E361G/D362G mutations (Arr-GG) had no effect on lactate production.

**Figure 6.**
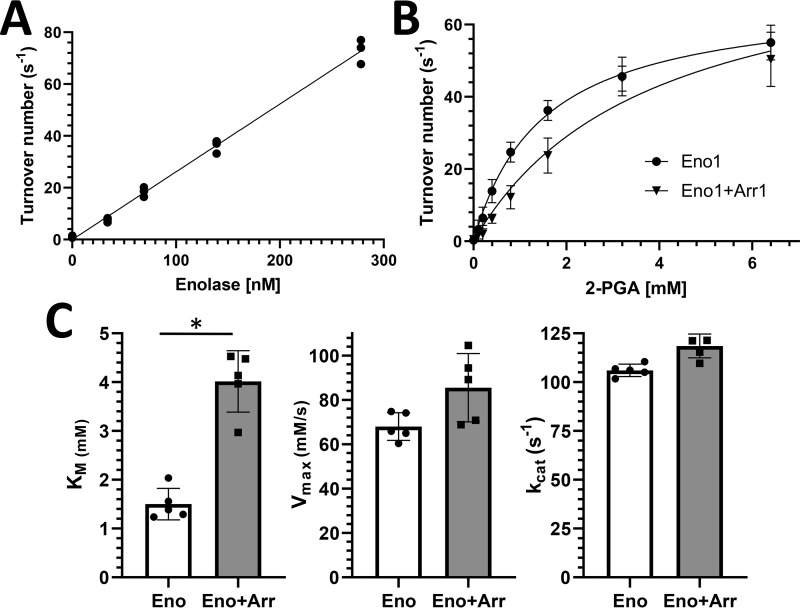
**Kinetic parameters for the effect of arrestin-1 on enolase-1 catalysis.** *A*, influence of enolase-1 concentration on turnover number for the catalysis of 2-PGA to PEP; the curve shows linear regression fit for replicate samples (*n* = 3). *B*, kinetics of the enolase-1 reaction for 2-PGA to PEP with 100 nm enolase-1 without arrestin-1 (●) or with 500 nm arrestin-1 (▾) in Michaelis–Menten plots; points show means ± S.D. (*n* = 5). Curves show nonlinear regression fit to Michaelis–Menten function. *C*, kinetic properties of enolase-1 with and without arrestin-1; *bars* represent mean ± S.D. (*error bars*) (*n* = 5) with statistically significant differences indicated with an *asterisk* (*p* < 0.05).

To gain a better understanding of how arrestin-1 affects the catalytic activity of enolase-1, we performed an analysis of enolase-1 under Michaelis–Menten conditions. We first determined the linear dependence of the 2-phosphoglycerate (2-PGA) to phosphoenolpyruvate (PEP) reaction rate on enolase-1 concentration (Fig. 6*A*). The kinetic parameters for this reaction was then measured using 100 nm enolase-1 with and without arrestin-1 (500 nm), varying the concentration of 2-PGA. A summary of the kinetic parameters (Fig. 6*C*) shows that the only significant effect of arrestin-1 was on the *K_m_*. The increase in *K_m_* without significant effects on either *V*_max_ or *k*_cat_ suggests that arrestin-1 acts as a competitive inhibitor, decreasing the access of the 2-PGA substrate for the active site in enolase-1.

## Discussion

This study provides several new points of important information regarding the arrestin-1/enolase-1 interaction. First, our quenching studies identified that arrestin-1 primarily engages enolase-1 along the surface opposite the side of the molecule that binds photoactivated rhodopsin. The concomitant binding of both enolase-1 and activated phosphorhodopsin supports this model. This observation suggests that in photoreceptors, when arrestin-1 translocates to the outer segment to bind pRho*, enolase-1 could be carried with the arrestin-1. However, this light-dependent movement of enolase-1 has not been observed (9), indicating that either the dissociation rate of enolase-1 from arrestin-1 is sufficiently high that enolase-1 is not being carried with arrestin-1 or enolase-1 might be interacting with other unidentified binding partners in the inner segment.

Another important conclusion that can be reached by the binding of arrestin-1 to both enolase-1 and pRho* is that the conformation of arrestin-1 that is bound by enolase-1 is independent of the known elements that regulate its transition from the inactive to the receptor-bound state. These regulatory elements include the “finger loop” of the N-terminal domain (24–27) and the phosphate sensors (24, 28). Our results showing equivalent binding of enolase-1 to the 3A mutant of arrestin-1 add further evidence that mobilization of the C terminus of arrestin-1 is not a critical regulatory element for enolase-1 binding either.

Our molecular model for the arrestin-1/enolase-1 complex, which was validated by targeted mutation of charge-pair interactions, permitted identification of the likely cause for arrestin's modulation of enolase-1 catalysis. In this model, Glu-361 and Asp-362 of arrestin-1 penetrate deeply into the enolase-1 protein. The proximity of these residues to loops L1, L2, and L3 (7.6, 4.9, and 6.0 Å, respectively), which comprise the main elements of the enolase-1 active site (21, 22), suggests that Glu-361/Asp-362 likely create a steric interference with the L1, L2, and L3 loops of enolase-1. This conclusion is supported by the results from the kinetic parameters investigation, which indicate that arrestin-1 acts as a competitive inhibitor.

The model for how arrestin-1 impacts enolase-1 activity also offers a potential explanation for the previous observation that arrestin-1 has a maximum effect of reducing enolase-1 activity by only ∼25% (9). Because arrestin-1 interacts with only one of the active sites in the enolase-1 dimer, the maximum effect that would be expected is only a 50% reduction in enolase activity, even if arrestin-1 completely disrupted the active site.

The functional consequences of the interaction between arrestin-1 and enolase-1 have not yet been elucidated. However, our empirical data from studying the glycolytic potential of kidney cells in tissue culture show that arrestin-1 can reduce the lactate output. This observation has some intriguing implications for photoreceptors, particularly given the very high glycolytic activity in rods and cones (12, 13). In these cells, which are highly modified and polarized sensory cilia, the inner segment portion of rods and cones is responsible for most of the metabolic activity of the photoreceptor, including glycolysis, whereas the outer segment is principally responsible for phototransduction. Accordingly, enolase-1 principally localizes to the inner segment of photoreceptors along with the other glycolytic enzymes (9, 29). In contrast, arrestin-1 localization is dynamic, translocating from the inner segment under dark conditions to the outer segment upon light exposure (*e.g.* see Refs. 30–33). This translocation of arrestin-1 means that arrestin-1 down-regulates the activity of enolase-1 during the dark, when it localizes to the inner segments, and that enolase-1 activity is up-regulated in the light when arrestin-1 moves to the outer segments. Because the energetic demands of photoreceptors are highest in the dark when the cyclic nucleotide–gated channels are open and the Na,K-ATPase is at its maximum rate to maintain ionic equilibrium (34), it is not clear what benefit might be provided by potentially increasing glycolytic activity in the light when enolase-1 quenching is reduced as arrestin-1 translocates to the outer segment. Perhaps an increase in glycolytic activity during light may function to provide more lactate to the RPE or Müller glia for their metabolic demands, rather than increasing the energy supply to photoreceptors. Regardless, this study's identification of the mechanism for how arrestin-1 impacts the activity of enolase-1 now opens the opportunity to empirically examine the functional effects of arrestin-1 on enolase-1 activity in photoreceptors and their surrounding cellular neighbors.

## Experimental procedures

### Arrestin-1 and enolase-1 mutagenesis

Cysteine substitution mutations, charge reversal mutations, and F375A/V376A/F377A mutations of arrestin-1 were introduced into N-terminally His_6_-tagged bovine arrestin-1 by overlapping PCR amplification of the bovine arrestin-1 cDNA as described previously (35). For the cysteine substitution mutants, the arrestin-1 cDNA template also contained the two cysteine mutations, C63A and C143A, to remove the two reactive cysteines that are endogenously present in arrestin-1 (27). Arrestin-1 containing combinations of mutants was prepared by serial mutagenesis, using the mutagenized cDNA as the template to add the next mutation, until all desired mutations were introduced.

The primers used to introduce these cysteine substitutions and charge reversal mutations are shown in Table 3. The altered cDNAs were cloned into pPICZ-A at the EcoRI site and heterologously expressed in *Pichia pastoris* for 3 days in the presence of 0.5% methanol. Following disruption of the cell wall by French pressing (20,000 p.s.i.), the arrestin-1 protein was purified to >95% homogeneity by chromatographic purification over nickel-agarose affinity resin (GE resin) in 50 mm sodium phosphate (pH 8.0) with 300 mm sodium chloride and 10 mm imidazole, eluting with 100 mm EDTA. Fractions containing purified arrestin-1 protein were pooled and dialyzed against LAP200^N^ buffer (50 mm HEPES, 200 mm NaCl, 1 mm EGTA, 1 mm MgCl_2_, 10% glycerol, 0.05% Nonidet P-40, pH 7.4).

**Table 3:** **Targeted mutations and primer pairs used for site-directed mutagenesis in arrestin-1** The arrestin-1 cDNA utilized contained an N-terminal His_6_ tag introduced after the initiating methionine and with C63A and C143A mutations to remove the two reactive cysteines.

Targeted substitution	Synthetic overlapping oligonucleotide pairs for mutagenesis
H10C	5′-AAGCCCGCACCAAACTGTGTTATCTTCAAGAAG
	5′-CTTCTTGAAGATAACACAGTTTGGTGCGGGCTT
R18C	5′-TTCAAGAAGATCTCCTGTGATAAATCGGTGACC
	5′-GGTCACCGATTTATCACAGGAGATCTTCTTGAA
Y25C	5′-GATAAATCGGTGACCATCTGTCTGGGGAAGAGAGATTAC
	5′-GTAATCTCTCTTCCCCAGACAGATGGTCACCGATTTATC
K28C	5′-GACCATCTACCTGGGGTGTAGAGATTACATAGAC
	5′-GTCTATGTAATCTCTACACCCCAGGTAGATGGTC
R37C	5′-ATAGACCACGTTGAATGTGTAGAGCCTGTGGATG
	5′-CATCCACAGGCTCTACACATTCAACGTGGTCTAT
E50C	5′-GTGCTGGTGGATCCATGTCTCGTGAAGGGCAAG
	5′-CTTGCCCTTCACGAGACATGGATCCACCAGCAC
K53C	5′-GATCCTGAGCTCGTGTGTGGCAAGAGAGTGTAC
	5′-GTACACTCTCTTGCCACACACGAGCTCAGGATC
I72C	5′-TACGGCCAGGAAGACTGTGACGTGATGGGC
	5′-GCCCATCACGTCACAGTCTTCCTGGCCGTA
S86C	5′-CAGGGACCTCTACTTCTGTCAAGTCCAGGTGTTCC
	5′-GGAACACCTGGACTTGACAGAAGTAGAGGTCCCTG
V94C	5′-CAGGTGTTCCCTCCATGTGGGGCCTCGGGCGCC
	5′-GGCGCCCGAGGCCCCACATGGAGGGAACACCTG
A113C	5′-ATCAAGAAGCTGGGCTGTAACACCTACCCCTTC
	5′-GAAGGGGTAGGTGTTACAGCCCAGCTTCTTGAT
Y125C	5′-CTCACGTTTCCTGACTGTTTGCCCTGTTCGGTG
	5′-CACCGAACAGGGCAAACAGTCAGGAAACGTGAG
V139C	5′-CCAGCTCCGCAAGATTGTGGCAAGAGCGCAGGG
	5′-CCCTGCGCTCTTGCCACAATCTTGCGGAGCTGG
K166C	5′-GAGGACAAAATTCCCTGTAAGAGCTCCGTGCG
	5′-CGCACGGAGCTCTTACAGGGAATTTTGTCCTC
D183C	5′-CAGCACGCGCCACGATGTATGGGTCCCCAGCCCCG
	5′-CGGGGCTGGGGACCCATACATCGTGGCGCGTGCTG
R189C	5′-GATATGGGTCCCCAGCCCTGTGCCGAGGCCTCCTGG
	5′-CCAGGAGGCCTCGGCACAGGGCTGGGGACCCATATC
W194C	5′-CGAGCCGAGGCCTCCTGTCAGTTCTTCATGTCG
	5′-CGACATGAAGAACTGACAGGAGGCCTCGGCTCG
S199C	5′-TGGCAGTTCTTCATGTGTGACAAGCCCCTGCGC
	5′-GCGCAGGGGCTTGTCACACATGAAGAACTGCCA
S210C	5′-CTCGCCGTCTCGCTCTGTAAAGAGATCTATTAC
	5′-GTAATAGATCTCTTTACAGAGCGAGACGGCGAG
E218C	5′-ATCTATTACCACGGGTGTCCCATTCCTGTGACC
	5′-GGTCACAGGAATGGGACACCCGTGGTAATAGAT
E231C	5′-GTGACCAACAGCACATGTAAGACAGTGAAGAAG
	5′-CTTCTTCACTGTCTTACATGTGCTGTTGGTCAC
S251C	5′-CGTGGTTCTCTACTGTAGTGATTATTAC
	5′-GTAATAATCACTACAGTAGAGAACCACG
K267C	5′-GAGGAAGCACAGGAATGTGTGCCGCCAAACAGC
	5′-GCTGTTTGGCGGCACACATTCCTGTGCTTCCTC
V281C	5′-AAGACGCTGACGCTGTGTCCCTTGCTGGCCAAC
	5′-GTTGGCCAGCAAGGGACACAGCGTCAGCGTCTT
E302C	5′-GGGAAAATCAAGCACTGTGACACGAACCTGGCC
	5′-GGCCAGGTTCGTGTCACAGTGCTTGATTTTCCC
D317C	5′-CATAAAGGAGGGAATATGTAAGACCGTCATGGGG
	5′-CCCCATGACGGTCTTACATATTCCCTCCTTTATG
D362C	5′-CATCCCCAGCCAGAGTGTCCAGATACCGCCAAG
	5′-CTTGGCGGTATCTGGACACTCTGGCTGGGGATG
E393C	5′-GCAGGAGAATATAAGTGTGAGAAGACAGACCAG
	5′-CTGGTCTGTCTTCTCACACTTATATTCTCCTGC
R29E	5′-CATCTACCTGGGGAAGGAAGATTACATAGACCAC
	5′-GTGGTCTATGTAATCTTCCTTCCCCAGGTAGATG
E36K	5′-GATTACATAGACCACGTTAAACGAGTAGAGCCTGTG
	5′-CACAGGCTCTACTCGTTTAACGTGGTCTATGTAATC
R37D	5′-CATAGACCACGTTGAAGATGTAGAGCCTGTGGATG
	5′-CATCCACAGGCTCTACATCTTCAACGTGGTCTATG
D183K	5′-CAGCACGCGCCACGCAAGATGGGTCCCCAGCC
	5′-GGCTGGGGACCCATCTTGCGTGGCGCGTGCTG
E302K	5′-GGGAAAATCAAGCACAAGGACACGAACCTGGCC
	5′-GGCCAGGTTCGTGTCCTTGTGCTTGATTTTCCC
E361K	5′-CATGCATCCCCAGCCAAAGGACCCAGATACCGCC
	5′-GGCGGTATCTGGGTCCTTTGGCTGGGGATGCATG
D362K	5′-CATCCCCAGCCAGAGAAGCCAGATACCGCCAAG
	5′-CTTGGCGGTATCTGGCTTCTCTGGCTGGGGATG
E361G/D362G	5′-CATGCATCCCCAGCCAGGAGGTCCAGATACCGCCAAGG
	5′-CCTTGGCGGTATCTGGACCTCCTGGCTGGGGATGCATG

Enolase-1 was prepared as described for arrestin-1 with the exception that the His_6_ tag was added at the C terminus. Fusion of enolase-1 with GFP was made by introducing an NheI restriction site prior to the stop codon in the enolase-1 cDNA with an His_6_ tag and then inserting the GFP cDNA flanked with NheI restriction sites. The fusion cDNA was cloned into pPIC-ZA and expressed and purified as described above for arrestin-1.

### Quenching assay

Cysteine mutants of arrestin-1 were labeled with mBBr (Thermo Fisher Scientific), reacting the arrestin-1 protein with a 100-fold molar excess of the fluorescent label for 2 h at room temperature. The unreacted label was removed by dialyzing against three sequential changes of 500 volumes of LAP200 buffer. Fluorescence labeling of the arrestin was quantified by measuring the absorbance of mBBr (394 nm, λ_max_) and arrestin-1 (278 nm, λ_max_) and calculating the percentage labeled (assuming *E*_MBB, 394 nm_ = 5,300 m^−1^ cm^−1^ and *E*_Arr1, 278 nm_ = 25,200 m^−1^ cm^−1^). Fluorescence quenching of the mBBr fluorophore was performed using 0.25 μm mBBr-labeled arrestin-1 with 100 mm potassium iodide in the presence or absence of 5 μm bovine enolase-1 that had been heterologously expressed and purified as described previously (9), monitoring fluorescence emission at 470 nm. To determine the degree to which enolase-1 shielded a residue from quenching by potassium iodide, we calculated a protection factor as follows,

(Eq. 1) $$P_{\text{f}}=\frac{F_{\left(\text{A}+\text{E}+\text{K}\right)}-F_{\left(\text{A}+\text{K}\right)}}{F_{\left(\text{A}\right)}-F_{\left(\text{A}+\text{K}\right)}}$$

where *F*_(A)_ is the fluorescence of mBBr-labeled arrestin in solution alone, *F*_(A + K)_ is the fluorescence of mBBr-arrestin-1 in the presence of potassium iodide, and *F*_(A + E + K)_ is the fluorescence of mBBr-arrestin-1 in the presence of both enolase-1 and potassium iodide. Essentially, this protection quotient looks at the total range of protection offered by enolase normalized to the access of the labeled cysteine to the potassium iodide. Values near 0 indicate no protection of the fluorophore-labeled cysteine, whereas the protection factor will approach 1 if enolase-1 completely shields the mBBr from quenching by the potassium iodide.

For quenching assays done in the presence of pRho*, pRho in disc membranes was sonicated (Sonic Dismembrator, Fisher) for 5 s in the dark to make a micellar preparation of the disc membranes. pRho (5 μm) was added to the samples and exposed to light for 1 min to activate the rhodopsin, and then fluorescence quenching measurements were made as described above.

### Molecular modeling

The interaction of arrestin-1 and enolase-1 was modeled using the crystallographic structure of arrestin-1 (1CF1, chain A (36)) and enolase-1 (3B97 (21)), using ClusPro 2.0 (37–39). Initial models were generated with no constraints, using arrestin-1 as the “receptor” molecule and enolase-1 as the “ligand.” In the highest-scored reported models, ClusPro 2.0 predicted many of the previously mentioned arrestin-1 residues (His-10, Asp-183, Glu-218, Glu-302, and Asp-362) as potential interface residues. To generate a more accurate prediction, the interaction sites were constrained to include the residues that showed the strongest protection of fluorescence quenching by enolase-1, namely His-10, Asp-183, Glu-218, Glu-302, and Asp-362.

### Rhodopsin disc membrane pulldown assay

Phosphorylated rhodopsin (pRho) in rod disc membranes was prepared as described previously (40). For pulldown, 2 μm arrestin-1 with or without 2 μm enolase-1/GFP was mixed with 5 μm pRho under dim red light. Samples with pRho* were activated by exposure to light for 1 min and then returned to the dark for processing. Samples were centrifuged for 15 min (18,000 × *g*), and the pellet was resuspended in Laemmli sample buffer (41) prior to separation on 12% SDS-gel electrophoresis and staining with 0.05% Coomassie R.

### Immunoprecipitation/enolase pulldown assay

The interaction between arrestin-1 and enolase-1 was assessed by using anti-arrestin-1 antibody to immunoprecipitate the arrestin-1–enolase-1 complex in which the enolase-1 was fluorescently labeled with Alexa Fluor 546. For this assay, 1.5 mg of Protein G–coated magnetic beads (DynaBeads, Thermo Fisher Scientific), were coated with 10 μg of purified C10C10 anti-arrestin-1 mAb (42). Enolase-1 was labeled on its reactive cysteines with Alexa Fluor 546 maleimide (Thermo Fisher Scientific), reacting the enolase-1 with a 100-fold molar excess of the fluorescent label. The fluorescently labeled enolase-1 was then sequentially dialyzed in LAP200^N^ buffer to remove any unreacted label. For the immunoprecipitation of the arrestin-1–enolase-1 complex, 5 μm arrestin-1 (or arrestin-1 mutant) was mixed with 5 μm enolase-1–Alexa 546 in LAP200^N^ in a 200-μl final volume for 2 h at 4 °C, to which 10 μg of anti-arrestin-1 antibody on magnetic beads was subsequently added for 16 h with gentle rotation. The beads were magnetically captured, washed 10 times with LAP200^N^ buffer, and then eluted with 0.1 m glycine (pH 2.5). After neutralizing the pH with 0.1 volume of 1.5 m Tris base (pH 8.5), the fluorescence of the captured enolase-1–Alexa 546 was measured, exciting fluorescence at 530 nm, and average emission at 570–575 nm was measured with a dual-detector fluorimeter (QM-1 steady state fluorescence spectrophotometer, Photon Technologies Inc.).

### Enolase activity assay

The catalytic activity of enolase-1 was measured in reconstitution assays by monitoring ATP production from the processing of phosphoenolpyruvate produced by enolase catalysis of 2-phosphoglycerate. For the reaction, 2 mm 2-phosphoglycerate (Sigma), 2 mm adenosine diphosphate (Sigma), and 0.3 units/ml pyruvate kinase (MP Biochemicals) was mixed with 50 nm enolase-1 with 0–1.6 μm arrestin-1 or arrestin-1 mutant. ATP production was monitored using a luciferase luminescence assay, mixing equal volumes of the reaction mixture and firefly luciferase (CellTiter-Glo 2.0 cell viability assay, Promega). Luminescence was monitored at 550–570 nm at 5-min intervals for 40 min, calculating the rate of luminescence production from a linear regression of luminescence as a measure of enolase catalytic activity.

For measuring glycolytic production of lactate in tissue culture, arrestin-1 (Arr-WT), arrestin-1 with E361G/D362G (Arr-GG), and GFP were cloned behind the ubiquitous small chicken β-actin (smCBA) promoter in the pTR-smCBA vector at the NotI sites (43). Human embryonic kidney cells (HEK-293T; 6 × 10^6^ cells) (44) were electroporated (Celetrix Biotechnologies), with 10 μg of each plasmid at 600 V for 3 ms and plated in 6-well tissue culture plates in Dulbecco's modified Eagle's medium with 5% fetal bovine serum. After 24 h, the medium was removed and replaced with fresh Dulbecco's modified Eagle's medium supplemented with 10 mm glucose and 1 μm rotenone and 1 μm antimycin A to block the respiratory chain (23). Medium samples were removed at 10-min intervals, and lactate was measured by luminescence according to the manufacturer's recommendation (Lactate-Glo, Promega).

For measuring the kinetic parameters of enolase-1 catalysis, enolase-1 activity was monitored by the increase in PEP absorbance at 240 nm using a ClarioStar plate reader (BMG Labtech) and UV-transparent 96-well plates. A linear dependence on the reaction was first determined using 0–300 nm enolase-1 with 2 mm 2-PGA (Sigma–Aldrich) in LAP200^N^ buffer. The turnover number was calculated using an extinction coefficient of 1,520 m^−1^ for PEP. Kinetic parameters under Michaelis–Menten conditions were then measured using 100 nm enolase-1 with 0–6 mm 2-PGA in LAP200^N^ buffer. Kinetic parameters *K_m_*, *V*_max_, and *k*_cat_ were estimated by GraphPad Prism (version 8.4; GraphPad Software).

### Statistical comparisons

Statistical comparisons were performed with GraphPad Prism. For multiple comparisons, one-way analysis of variance was conducted with Sidak's post hoc multiple comparisons.

For enolase catalysis, the effect of arrestin-1 on enolase-1 activity was curve-fit by nonlinear regression analysis (inhibitor *versus* response). Rate of lactate production from HEK-293T cells was determined from the slope of a best-fit linear regression. In all cases, differences were considered significant if *p* was <0.05.

### Data availability

All data for this publication are either included in the article or available from the corresponding author upon request.

## Author contributions

C. J. M. and W. C. S. conceptualization; C. J. M. and W. C. S. resources; C. J. M. and W. C. S. data curation; C. J. M., J. T. A., R. M., and W. C. S. formal analysis; C. J. M., R. M., and W. C. S. supervision; C. J. M. and W. C. S. funding acquisition; C. J. M., N. F., N. K., D. T., D. B., S. N. B., J. T. A., R. M., and W. C. S. investigation; C. J. M., J. T. A., R. M., and W. C. S. methodology; C. J. M. and W. C. S. writing-original draft; C. J. M. and W. C. S. project administration; C. J. M., N. F., N. K., D. T., D. B., S. N. B., J. T. A., R. M., and W. C. S. writing-review and editing.

## Footnotes

4 The abbreviations used are:

NSF: *N*-ethylmaleimide–sensitive factor
mBBr: monobromobimane
pRho: phosphorylated rhodopsin
pRho*: light-activated phosphorylated rhodopsin
2-PGA: 2-phosphoglycerate
PEP: phosphoenolpyruvate.
